# Dental plaque microbiota sequence counts for microbial profiling and resistance genes detection

**DOI:** 10.1007/s00253-024-13152-z

**Published:** 2024-05-06

**Authors:** Laura Veschetti, Salvatore Paiella, Maria Carelli, Francesca Zotti, Erica Secchettin, Giuseppe Malleo, Caterina Signoretto, Giorgia Zulianello, Riccardo Nocini, Anna Crovetto, Roberto Salvia, Claudio Bassi, Giovanni Malerba

**Affiliations:** 1 Department of Neurosciences, Biomedicine and Movement Sciences, University of Verona, Verona, Italy; 2 General and Pancreatic Surgery Unit, Pancreas Institute, University of Verona, Verona, Italy; 3 Department of Surgical Sciences, Dentistry, Gynaecology and Paediatrics, University of Verona, Verona, Italy; 4 Department of Diagnostics and Public Health, University of Verona, Verona, Italy

**Keywords:** Shotgun metagenomics, Sequencing depth, Antimicrobial resistance, Experimental design

## Abstract

**Abstract:**

Shotgun metagenomics sequencing experiments are finding a wide range of applications. Nonetheless, there are still limited guidelines regarding the number of sequences needed to acquire meaningful information for taxonomic profiling and antimicrobial resistance gene (ARG) identification. In this study, we explored this issue in the context of oral microbiota by sequencing with a very high number of sequences (~ 100 million), four human plaque samples, and one microbial community standard and by evaluating the performance of microbial identification and ARGs detection through a downsampling procedure. When investigating the impact of a decreasing number of sequences on quantitative taxonomic profiling in the microbial community standard datasets, we found some discrepancies in the identified microbial species and their abundances when compared to the expected ones. Such differences were consistent throughout downsampling, suggesting their link to taxonomic profiling methods limitations. Overall, results showed that the number of sequences has a great impact on metagenomic samples at the qualitative (i.e., presence/absence) level in terms of loss of information, especially in experiments having less than 40 million reads, whereas abundance estimation was minimally affected, with only slight variations observed in low-abundance species. The presence of ARGs was also assessed: a total of 133 ARGs were identified. Notably, 23% of them inconsistently resulted as present or absent across downsampling datasets of the same sample. Moreover, over half of ARGs were lost in datasets having less than 20 million reads. This study highlights the importance of carefully considering sequencing aspects and suggests some guidelines for designing shotgun metagenomics experiments with the final goal of maximizing oral microbiome analyses. Our findings suggest varying optimized sequence numbers according to different study aims: 40 million for microbiota profiling, 50 million for low-abundance species detection, and 20 million for ARG identification.

**Key points:**

*• Forty million sequences are a cost-efficient solution for microbiota profiling*

*• Fifty million sequences allow low-abundance species detection*

*• Twenty million sequences are recommended for ARG identification*

**Graphical Abstract:**

**Figure:**
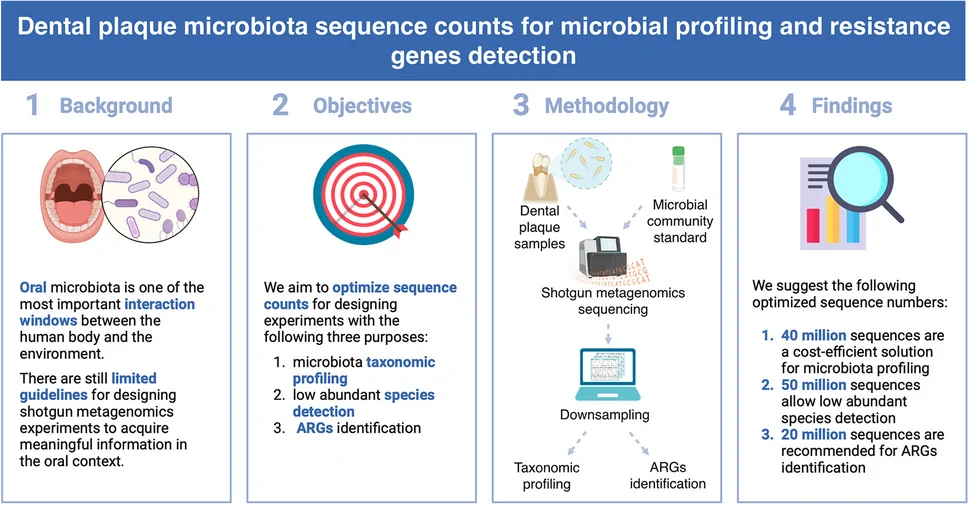

**Supplementary Information:**

The online version contains supplementary material available at 10.1007/s00253-024-13152-z.

## Introduction

Microbiota is known as the entire set of microorganisms—comprising bacteria, archaea, viruses, and eukaryotes—present in a defined *niche* (Berg et al. 2020). Its composition and functions can be explored through a variety of methods, the most widespread ones being 16S rRNA gene sequencing and shotgun metagenomics. The former is also known as metabarcoding or metataxonomics and consists of the targeted sequencing of 16S rRNA gene hypervariable regions. Such an approach allows to obtain an overview of the bacterial community under study by estimating its taxonomical composition starting from a relatively small number of sequences (18,000–30,000 sequences per sample) (Kozich et al. 2013). In particular, metabarcoding analyses allow to gain insights into microbial community diversity and richness and draw comparisons between different *niches* or sample types; nonetheless, little to no information can be collected regarding the functional potential and activity of the communities. Moreover, it has been reported that the choice of primers used during sample preparation could lead to potential biases in the representation of some taxonomic groups (Campanaro et al. 2018) and that some taxa—especially less abundant ones—could be lost (Durazzi et al. 2021).

Shotgun metagenomics overcomes such shortcomings by sequencing all the genetic content (i.e., sampling genes from all microbial genomes) retrievable from a defined *niche* (i.e., untargeted sequencing). This method has recently found a wide range of applications that encompass human health (Rampelli et al. 2020), ecological *niches* characterization (Loza et al. 2022), and public health risk assessment, with a particular focus on antimicrobial resistance reservoirs (Rubiola et al. 2022). Indeed, antimicrobial resistance is currently a cause of growing concern, and many fear a “post-antibiotic era” in which even common infections could become life-threatening (Noyes et al. 2016). This highlights the need to understand and characterize the mechanisms underlying antimicrobial resistance and calls for attentive and coordinated monitoring of resistance reservoirs worldwide (Mader et al. 2022). Shotgun metagenomics sequencing allows to analysis of the entire set of antimicrobial resistance genes (ARGs) carried by all microorganisms in a sample: this approach might improve the understanding of how and where resistance develops and spreads, as well as the discovery and characterization of still unknown resistance determinants.

However, this approach has a much higher cost than metabarcoding due to the need for a greater number of sequences (millions of sequences per sample) and produces high-complexity datasets that require extensive expertise to be analyzed (Quince et al. 2017), and there are still limited guidelines regarding the number of sequences needed to acquire meaningful information from shotgun metagenomics sequencing datasets, especially regarding ARGs identification.

Molecular studies have recently enabled researchers to dissect the complexity of microbiota composition and metabolic potential in different anatomical *niches* (Integrative HMP (iHMP) Research Network Consortium 2019). Among them, the oral cavity is drawing the attention of the scientific community as one of the most important interaction windows between the human body and the environment. Oral microbiota—with more than 700 species identified—is one of the most diverse microbial communities in the human body, and different microbial profiles have been associated with systemic diseases and cancer (Peng et al. 2022; Tuominen and Rautava 2021). In the present work, we analyzed the oral microbiota from four individuals that were investigated with a very high number of sequences (~ 100 million each), and we evaluated the performance of microbial identification and ARG detection through a downsampling procedure (i.e., randomly discarding fractions of sequences). Thus, we gained knowledge that can help design shotgun metagenomics experiments that are cost-efficient (i.e., to obtain the maximum useful information with the minimum cost possible) and suitable for the intended purposes in the oral microbiome context.

## Methods

### Sample collection and sequencing

Dental plaque samples were collected from four patients followed at Verona Hospital who were 18–75 years of age, did not receive antimicrobial therapy in the 4 weeks preceding sampling, did not wear mobile dentures or prosthetic dental appliances, and did not have active smoking or alcohol habits, dietary disorders, immune system disorders, or diabetes. Sampling collection of subgingival plaque was carried out using a sterile periodontal curette. The collected samples were placed in a 1.5-ml sterile centrifugal tube containing RNAlater solution (QIAGEN GmbH, Hilden, Germany), immediately transported to the laboratory, and centrifuged at 12,000 g for 15 min at 4 ℃. Genomic DNA was extracted within 1 h from the collection using the QIAamp DNA Blood Mini Kit (Qiagen, Milan, Italy) according to the manufacturer’s instructions. DNA was eluted in 100 µL double-distilled water and temporally stored at − 20 °C. The quality of extracted DNA was assessed using Qubit (Thermo Fisher Scientific, Wilmington, DE, USA) and Fragment Analyzer System (Agilent Technologies, Santa Clara, CA, USA). As a sequencing quality control, a Microbial Community DNA Standard (Zymo Research, Irvine, CA, USA) was processed together with the samples starting from the library generation step. The theoretical composition of the microbial community standard comprises 10 species with the following abundances: *Pseudomonas aeruginosa* (6.1%), *Escherichia coli* (8.5%), *Salmonella enterica* (8.7%), *Lactobacillus fermentum* (21.6%), *Enterococcus faecalis* (14.6%), *Staphylococcus aureus* (15.2%), *Listeria monocytogenes* (13.9%), *Bacillus subtilis* (10.3%), *Saccharomyces cerevisiae* (0.57%), and *Cryptococcus neoformans* (0.37%). Sequencing libraries were prepared using the KAPA PCR-free kit (Roche Sequencing Solutions, Pleasanton, CA, USA). All samples underwent shotgun metagenomic sequencing at the Technological Platform Centre of the University of Verona on a NextSeq500 Illumina platform (Illumina, Hayward, CA, USA) generating 150-bp paired-end reads.

### Sequencing data downsampling and quality controls

**Fig. 1:**
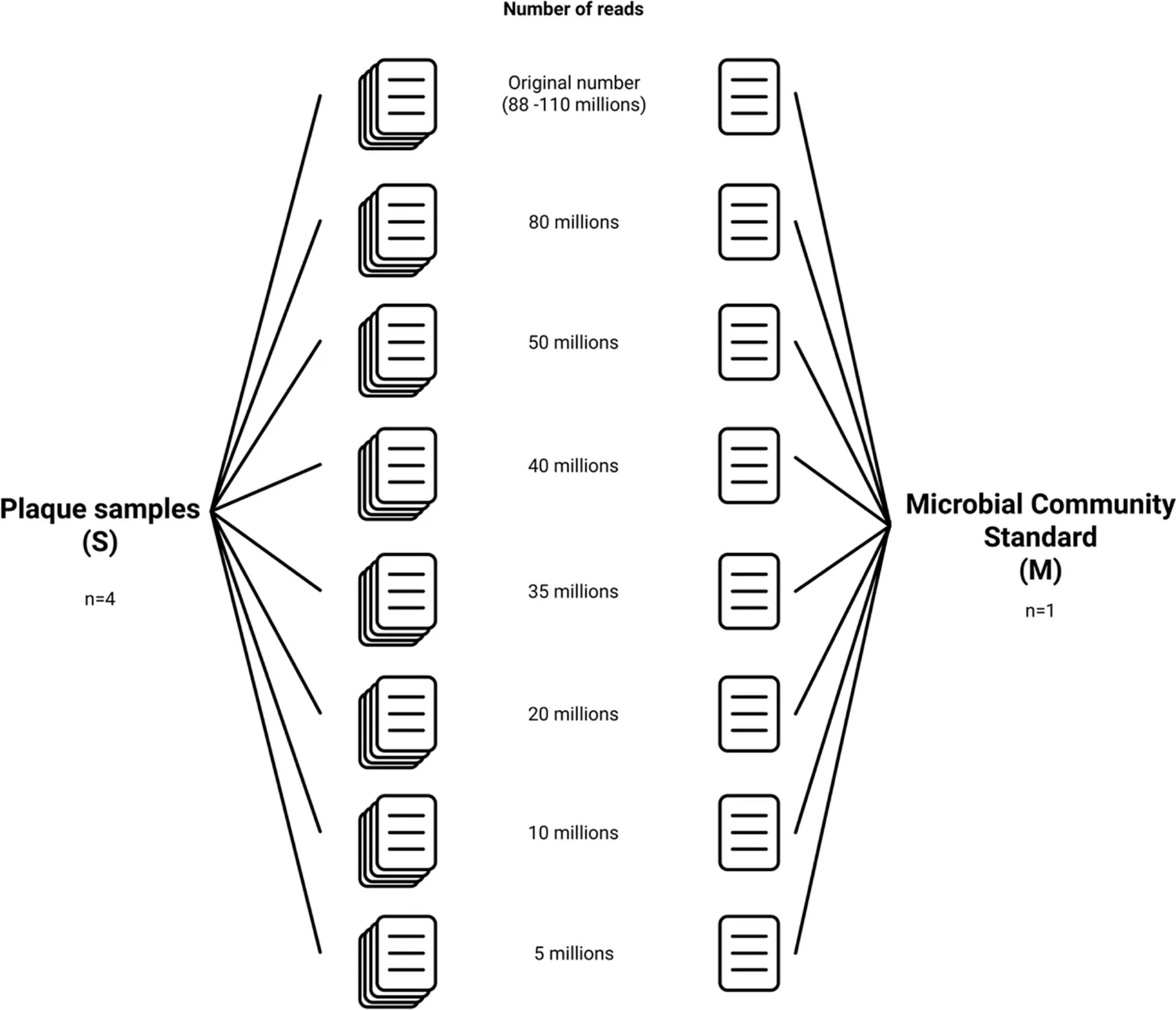
Schematic representation of sequencing data downsampling and dataset generation. Raw sequences (reads) of plaque samples and the microbial community standard were downsampled by generating 8 datasets with a decreasing number of sequences for each DNA sample

Raw reads of plaque samples (*n* = 4) and of the microbial community standard (*n* = 1) were downsampled obtaining a total of eight datasets of five samples each characterized by a decreasing number of sequences (Fig. 1). In particular, eight datasets were generated for each DNA sample by selecting the first *N* sequences in the FASTQ files. Datasets with the following number of sequences were generated: original number (range, 88–110 million sequences), 80, 50, 40, 35, 20, 10, and 5 million sequences. Each dataset was labeled according to the following nomenclature: one letter among S or M (*S* = sample, *M* = mock microbial community standard), identification number, underscore, number of million sequences in the dataset, e.g., S2_40M indicates the dataset from sample 2 with 40 million sequences (see Supplementary Table S1 for a list of all generated datasets). Quality controls of sequencing data were performed using the KneadData tool (available at https://github.com/biobakery/kneaddata) with default settings; briefly, the quality of raw reads was assessed using FastQC v0.11.9 (Andrews 2010), and adapter and base quality trimming was performed accordingly with Trimmomatic v0.39 (Bolger et al. 2014) using the following parameters: *Illuminaclip:adapter_file.fa:2:30:20 leading:3 trailing:3 slidingwindow:4:20 minlen:50*. Reads were then aligned to the *Homo sapiens* (human) genome GRCh38 using bowtie2 v2.3.5.1 (Langmead and Salzberg 2012) for host DNA contamination removal (i.e., sequence mapping to the human genome were discarded from further analyses).

### Quantitative taxonomic profiling

Sequencing data was analyzed with the MetaPhlAn3 v3.1.0 tool (Beghini et al. 2021) for profiling the communities’ composition (Bacteria, Archaea, and Eukaryotes). The quantitative profiling was obtained using bowtie2 to map reads against the CHOCOPhlAn v30 database for taxonomic classification, which comprehends ~ 1.1 M unique clade-specific marker genes identified from ~ 100,000 reference genomes (~ 99,500 bacterial and archaeal and ~ 500 eukaryotic). In particular, taxonomic profiling relies on detecting the presence and estimating the coverage of a collection of species–specific marker genes to estimate the relative abundance of known and unknown microbial taxa in shotgun metagenomic samples. Graphs and figures were generated using ggplot2 and fmsb packages in R v4.2.1 (R Core Team 2021).

### ARGs identification

ARGs identification was performed using bwa the v0.7.17-r1188 (Li and Durbin 2009) to align microbial sequences to MEGARes 2.0 (Doster et al. 2020) database (downloaded on 1st August 2022, *n* = 6635 nucleotide sequences), which contains antimicrobial drugs, biocides, and metal resistance determinant sequences. A Java-based script developed by Noyes et al. (2016) (available at: https://github.com/colostatemeg/gene_fraction_script/releases) was used to parse the resulting SAM files such that for each ARG identified in each sample, the proportion of nucleotides in the MEGARes ARG sequence that aligned with at least one read was calculated. In order to decrease the number of false positive ARG identifications (Gibson et al. 2015), only ARGs with > 50% of nucleotides covered by at least one read were defined as present in the sample and included in subsequent analyses. Graphs and figures were generated using ggplot2, forcats, and pheatmap packages in R v4.2.1 (R Core Team 2021).

## Results

### Sequencing and downsampling

**Fig. 2:**
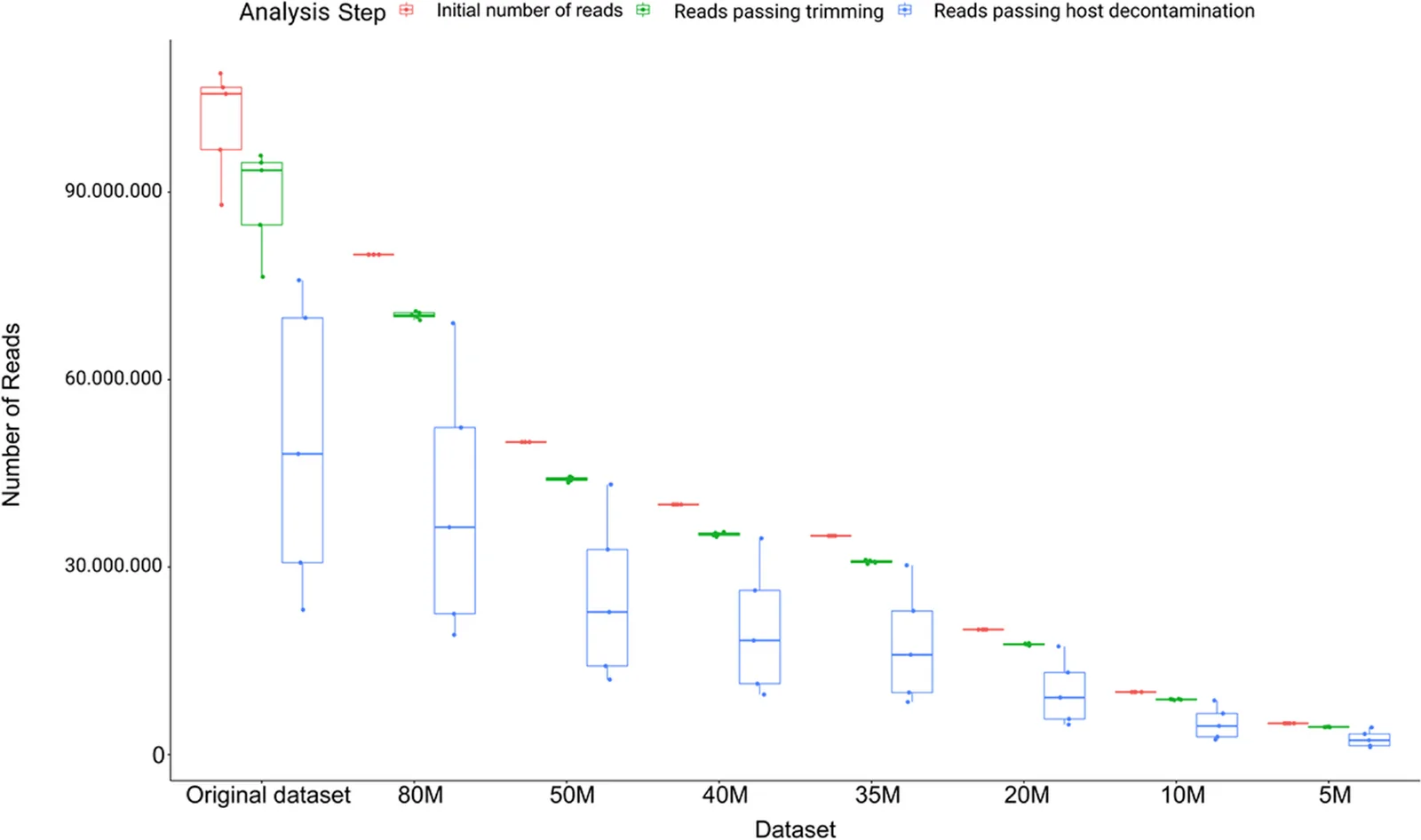
Quality controls results. Boxplot of the number of reads of the analyzed datasets at each quality control step: The number of reads passing adapter and quality trimming is reported in green, whereas reads passing host decontamination are reported in blue. Host decontamination was performed by removing reads mapping to the human genome (GRCh38)

The sequencing run yielded a mean of 101,241,917 reads (range = 87,950,201–109,004,045) per sample, and downsampling datasets containing 80, 50, 40, 35, 20, 10, and 5 million sequences were generated (Fig. 2). Considering all generated datasets, on average 88% of the reads (range = 87–89%) passed base quality and adapter trimming whereas 50% of the total number of reads (range = 28–87%) passed the subsequent host decontamination step (Supplementary Table S2). Overall, the downsampling datasets conserved the characteristics of the original dataset in terms of GC content, average read length, and proportion of reads passing each step of the quality control and decontamination procedure.

### Taxonomic profiling

**Fig. 3:**
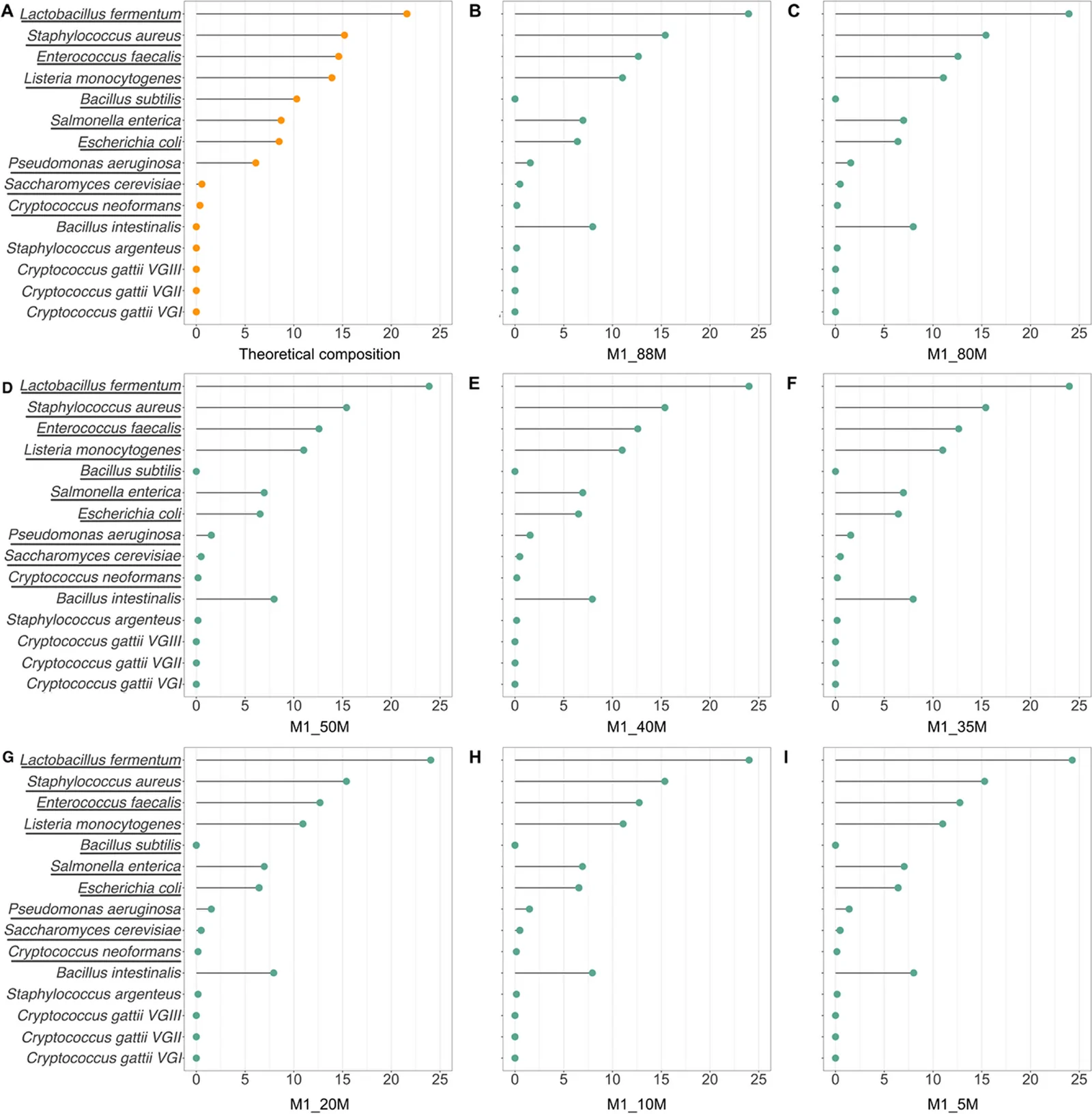
Microbial community standard quantitative taxonomic profiling. The *x*-axis indicates percent abundance and underlined names indicate species present in the microbial community standard composition declared in the product datasheet. **A** Theoretical microbial community composition as reported in the product datasheet. **B** Original shotgun metagenomics dataset comprising 88 million sequences. **C–I** Downsampling datasets including 80, 50, 40, 30, 20, 10, and 5 million sequences, respectively

Firstly, we investigated the impact of downsampling on quantitative taxonomic profiling in the M1 datasets (microbial community standard), since the real composition of the sample—both in terms of microorganisms and abundances—is known and reported in the product datasheet (Fig. 3). The theoretical composition of M1 (Fig. 3A) comprises 10 species, namely *Pseudomonas aeruginosa*, *Escherichia coli*, *Salmonella enterica*, *Lactobacillus fermentum*, *Enterococcus faecalis*, *Staphylococcus aureus*, *Listeria monocytogenes*, *Bacillus subtilis*, *Saccharomyces cerevisiae*, and *Cryptococcus neoformans*. When comparing the theoretical composition with the original shotgun metagenomics sequencing dataset (M1_88M) profiling—which is the one with the highest sequencing depth—we noticed some differences in the identified species. In particular, some *S. aureus* sequences were identified as *S. argenteus*, *B. subtilis* reads were entirely classified as *Bacillus intestinalis*, some *C. neoformans* sequences were reported as belonging to *Cryptococcus gattii*, and a lower abundance of *P. aeruginosa* species was reported (Supplementary Table S3).

**Fig. 4:**
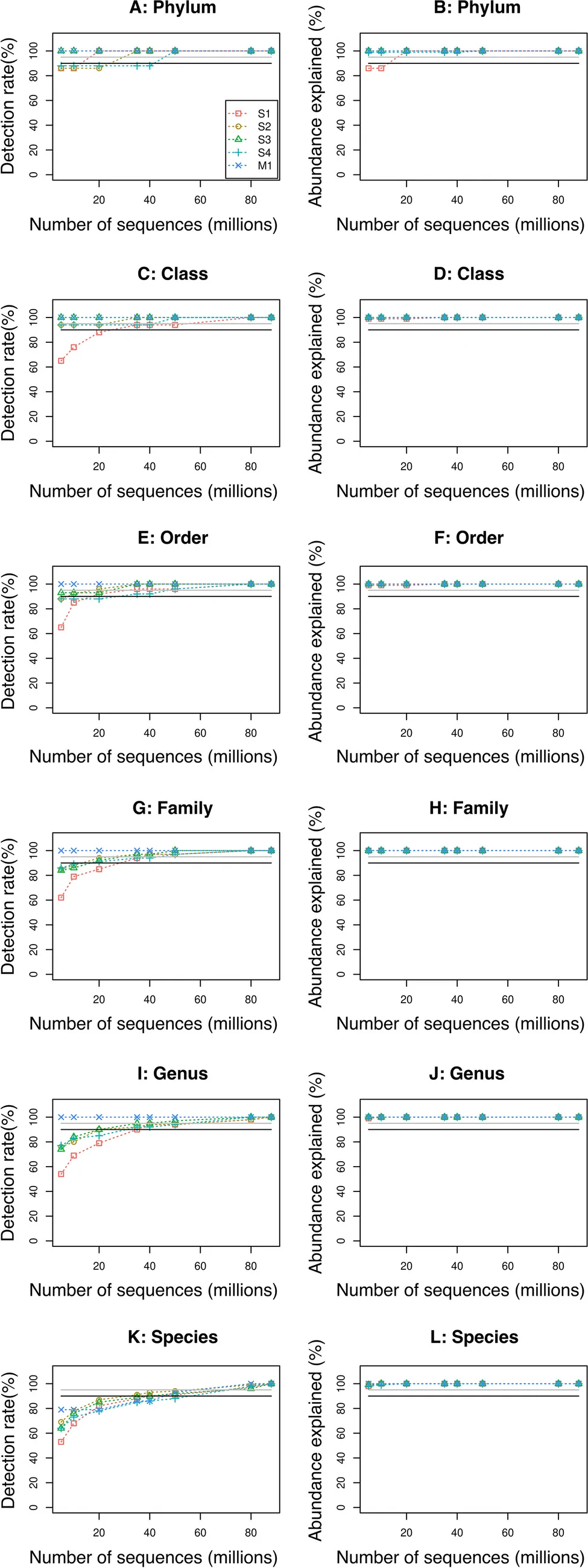
Taxonomic profiling of human plaque samples datasets. The detection rate was calculated as the ratio of identified classes in subsampling datasets at each taxonomic rank to the number of identified classes in the highest sequence number datasets. The abundance explained was also calculated keeping as reference the highest sequence number datasets. Black and grey lines indicate 90% and 95% thresholds, respectively

Since microbial community standards are simple communities composed of few and abundant species and do not reflect the complexities and difficulties of real metagenomic samples analysis, we performed qualitative taxonomic profiling (i.e., evaluation of the presence or absence of microorganisms’ taxa) and compared results across four downsampling datasets with decreasing number of sequences of four human plaque samples. Overall, we identified a mean number of 160 species (range, 133–191), 53 *genera* (range, 48–62), 35 families (range, 33–37), 26 orders (range, 25–27), 17 classes (range, 16–18), and 8 *phyla* (range,7–8) (Supplementary Table S4). The results (Fig. 4, left column) show that a decreasing number of sequences has a small impact on the number of identified classes at each taxonomic level in the M1 dataset, whereas it has a great impact on real samples (S1–4). Indeed, when comparing the original sequencing dataset of each sample with the corresponding 5-million sequence dataset, we observe a loss of information: a median value of 1 (range, 0–1) *phylum*, 1 (range, 0–6) class, 3 (range, 2–9) orders, 6 (range, 5–13) families, 14 (range, 12–22) *genera*, and 62 (range, 45–69) species are lost. In particular, to achieve a 90% detection rate at the species level in three out of four samples, at least 40 million sequences are required, whereas to achieve the same in all samples, 50 million sequences are needed (Fig. 4K). Moreover, to achieve a 95% detection rate in all samples at least 80 million sequences are necessary. Noticeably, S1 shows a more extreme loss in detection rate at less than 40 million reads when compared to other samples. This is due to the fact that S1 is taxonomically less diverse than the other samples; consequently, the loss of a single taxon has a marked impact on the detection rate as calculated.

Additionally, we investigated how a decreasing number of sequences affects abundance estimation at different taxonomic ranks, i.e., whether the abundance estimation of microorganisms changed within datasets (Fig. 4, right column). Overall, a small information loss (< 1% of total abundance) was observed at every taxonomic level, and variable abundance values were detected at *phylum*, class, order, and family levels in datasets having less than 35 million sequences (Supplementary Figures S1-S5). Moreover, results show variable abundance values at *genus* and species levels for low-abundance microorganisms (abundance < 0.1%) in datasets having less than 50 million sequences.

### ARGs identification

**Fig. 5:**
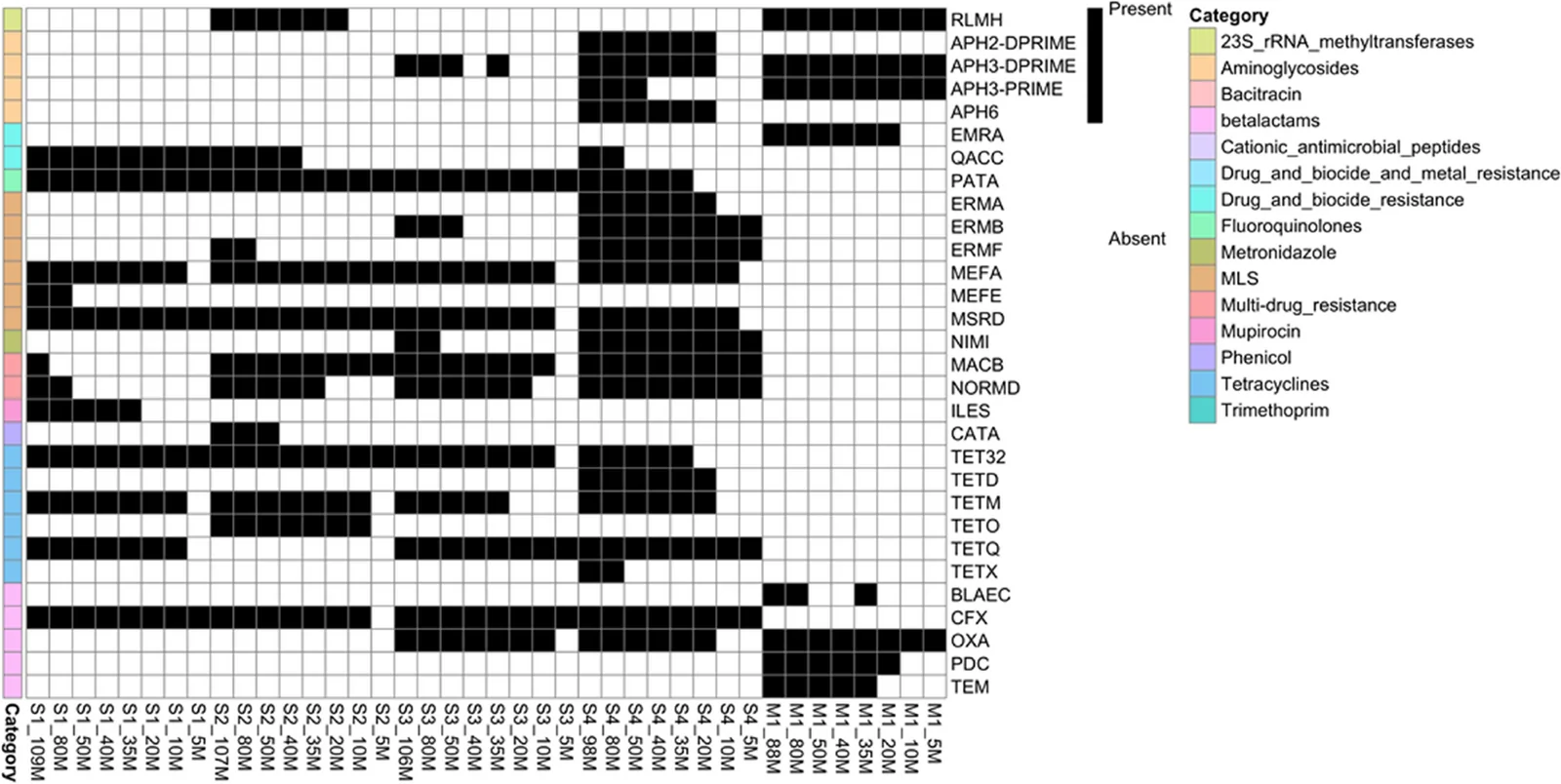
Antimicrobial resistance genes (ARGs) presence/absence heatmap. Only ARGs that did not consistently result as present or absent across different downsampling datasets of the same sample are shown for readability purposes. Only ARGs with > 50% of nucleotides covered by at least one read were defined as present in the sample. MLS macrolides, lincosamides, streptogramines

The presence of ARGs was assessed in each dataset: a total of 133 ARGs were identified (Supplementary Figure S6). Notably, 23% (*n* = 30) of them inconsistently resulted as present or absent across different downsampling datasets of the same sample (Fig. 5). In particular, we observed that the information loss increased with a diminishing number of sequences: at 80 million sequences, 1 ARG was lost; at 50 million 8 ARGs; at 40 million, 12 ARGs; at 35 million, 11 ARGs; at 20 million, 19 ARGs; at 10 million, 29 ARGs; and at 5 million sequences, 42 ARGs were lost. Overall, we observed that more than half of ARGs were lost in datasets having less than 20 million sequences.

## Discussion

In the present work, we evaluated the performance of microbial identification and ARG detection through a downsampling procedure in order to optimize sequence counts for designing shotgun metagenomics experiments that are cost-efficient and suitable for the intended purposes in the oral microbiome context. Raw reads of human plaque samples and of the microbial community standard were downsampled obtaining a total of eight datasets of five samples each characterized by a decreasing number of sequences. Overall, the downsampling datasets conserved the characteristics of the original dataset in terms of GC content, average read length, and proportion of reads passing each step of the quality control and decontamination procedure. Thus, the generated datasets were deemed suitable to perform a comparison and investigate the impact of decreasing the number of sequences on microbial identification and ARG detection.

Since the real composition of the microbial community standard—both in terms of microorganisms and abundances—is known, we first focused on quantitative taxonomic profiling of these datasets. When comparing the theoretical composition with the profiling results, we found some discrepancies regarding both the identified species and their estimated abundance. Of note, such differences are consistent across the different downsampling datasets, suggesting that they are linked to current taxonomic profiling methods rather than the number of sequences. This finding is in contrast with the widespread notion that shotgun metagenomics typically yields a detailed taxonomic resolution, even at species and strain levels (Truong et al. 2017), and underlines the need for improved taxonomic profiling tools and more comprehensive databases for microbial species and strains classification.

Microbial community standards, moreover, are simple communities composed of few abundant species and do not reflect the complexities of microorganisms’ communities found in real samples. A suggestive example was recently given by Kennedy and Chang (2020), who reported a great variability in microorganism communities’ richness and taxonomic profile, even “just” focusing on changes across different human body sites. Given the increasing scientific interest in the study of the human oral microbiota, we expanded our analysis by including human plaque samples collected from four individuals. Overall, a mean number of 160 species (range, 133–191), 53 *genera* (range, 48–62), 35 families (range, 33–37), 26 orders (range, 25–27), 17 classes (range, 16–18), and 8 *phyla* (range,7–8) have been identified, which is concordant with the expected number of microorganisms found in oral microbiota (Caselli et al. 2020; Dewhirst et al. 2010). In contrast to the microbial community standard, in which sequence downsampling has a small impact on qualitative taxonomic profiling, the more complex plaque samples require a higher number of sequences for a reliable taxonomic picture. Hence, the expected richness and diversity of microbial communities in the samples under analysis are to be taken into consideration when designing shotgun metagenomics experiments. Additionally, for plaque samples, 40 million sequences appear to be a good sequence count to obtain useful information for oral microbiota profiling. Indeed, with 40 million sequences, 10% of the identified species are lost, but they account for only < 1% of overall abundance. Of note, to increase the species detection rate from 90 to 95%, the number of sequencing reads needs to double from 40 to 80 million sequences.

In plaque sample datasets with more than 50 million sequences, low-abundance species (abundance < 0.1%) have a good chance of being still detected at *genus* and species levels. These findings enforce results reported in previous literature highlighting sensitivity problems in shotgun metagenomics experiments when dealing with low (< 2%) and very low (< 1%) abundant species (Pereira-Marques et al. 2019). Overall, 50 million sequences for plaque samples appear to be a good number of sequences to obtain a reliable abundance overview. Nonetheless, a much higher number of sequences is needed if one of the aims of the experiment is to detect and characterize low-abundance species.

Finally, we explored the impact of sequence downsampling on ARG identification, which is one of the most relevant and impactful applications of shotgun metagenomics. Overall, 23% of identified ARGs were not consistently present across different downsampling datasets of the same sample, with more than half of them being undetected below 20 million sequences. Thus, for plaque samples, a threshold of 20 million sequences seems reasonable to design a cost-effective experiment. These results indicate that shotgun metagenomics is a very promising approach to investigating antimicrobial resistance development and dissemination, as some examples in the literature corroborate (Noyes et al. 2016).

Even though we gained precious insights into oral microbiota shotgun metagenomics experimental designs, the current study presents some limitations. Among them, we analyzed a limited number of samples. Nevertheless, their composition fit with the expected number of microorganisms found in oral microbiota indicating that our results could suggest generalizable guidelines. Moreover, we did not take into consideration varying degrees of host DNA; indeed, plaque samples present a host DNA contamination of around 35–45%. However, the impact of varying degrees of host DNA contamination has been recently deeply explored by Pereira-Marques and colleagues (2019).

In conclusion, this study highlights the importance of carefully considering sequencing aspects and suggests some guidelines for designing shotgun metagenomics experiments with the final goal of maximizing oral microbiome analyses. Our findings suggest varying optimized sequence numbers according to different study aims: 40 million for microbiota profiling, 50 million for low-abundance species detection, and 20 million for ARG identification.

## Supplementary Information

Below is the link to the electronic supplementary material.

Supplementary file1 (XLSX 30.3 KB)

Supplementary file2 (PDF 2.58 MB)

## Data Availability

Sequencing data were submitted to the ENA database with project number PRJEB67641.
