# Supplementary material for: Dental plaque microbiota sequence counts for microbial profiling and resistance genes detection
Source: Appl Microbiol Biotechnol. 2024 May 6;108(1):319. doi: 10.1007/s00253-024-13152-z (PMC11074014; doi:10.1007/s00253-024-13152-z)
Supplement: Supplementary file 2 — Supplementary file2 (PDF 2.58 MB) [file 253_2024_13152_MOESM2_ESM.pdf]

Dental Plaque Microbiota Sequence Counts for Microbial Profiling and Resistance Genes Detection

Applied Microbiology and Biotechnology

Laura Veschetti[1], Salvatore Paiella[2], Maria Carelli[4], Francesca Zotti[3], Erica Secchettin[2], Giuseppe Malleo[2], Caterina Signoretto[4], Giorgia Zulianello[2], Riccardo Nocini[3], Anna Crovetto[2], Roberto Salvia[2], Claudio Bassi[2†], Giovanni Malerba[3]

[1] Department of Neurosciences, Biomedicine and Movement Sciences, University of Verona, Verona, Italy
[2] General and Pancreatic Surgery Unit, Pancreas Institute, University of Verona, Verona, Italy
[3] Department of Surgical Sciences, Dentistry, Gynaecology and Paediatrics, University of Verona, Verona, Italy
[4] Department of Diagnostics and Public Health, University of Verona, Verona, Italy

* Email of corresponding author: giovanni.malerba@univr.it
[†] Deceased

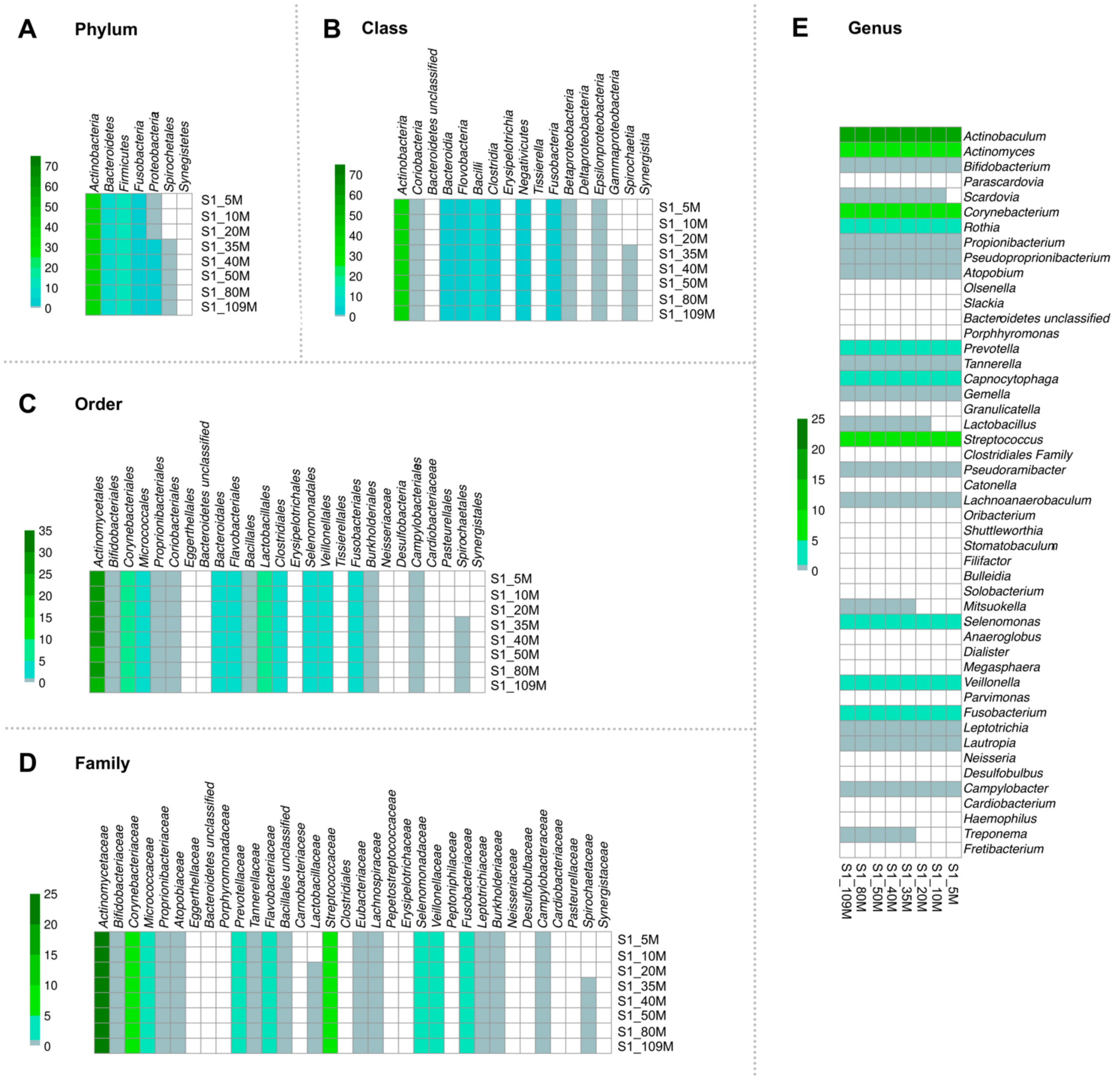

Supplementary Figure S1. Quantitative taxonomic profiling of S1datasets at different taxonomic levels: **A** *Phylum*, **B** Class, **C** Order, **D** Family, and **E** *Genus*. The colored bar (grey to dark green) indicates percent abundance.

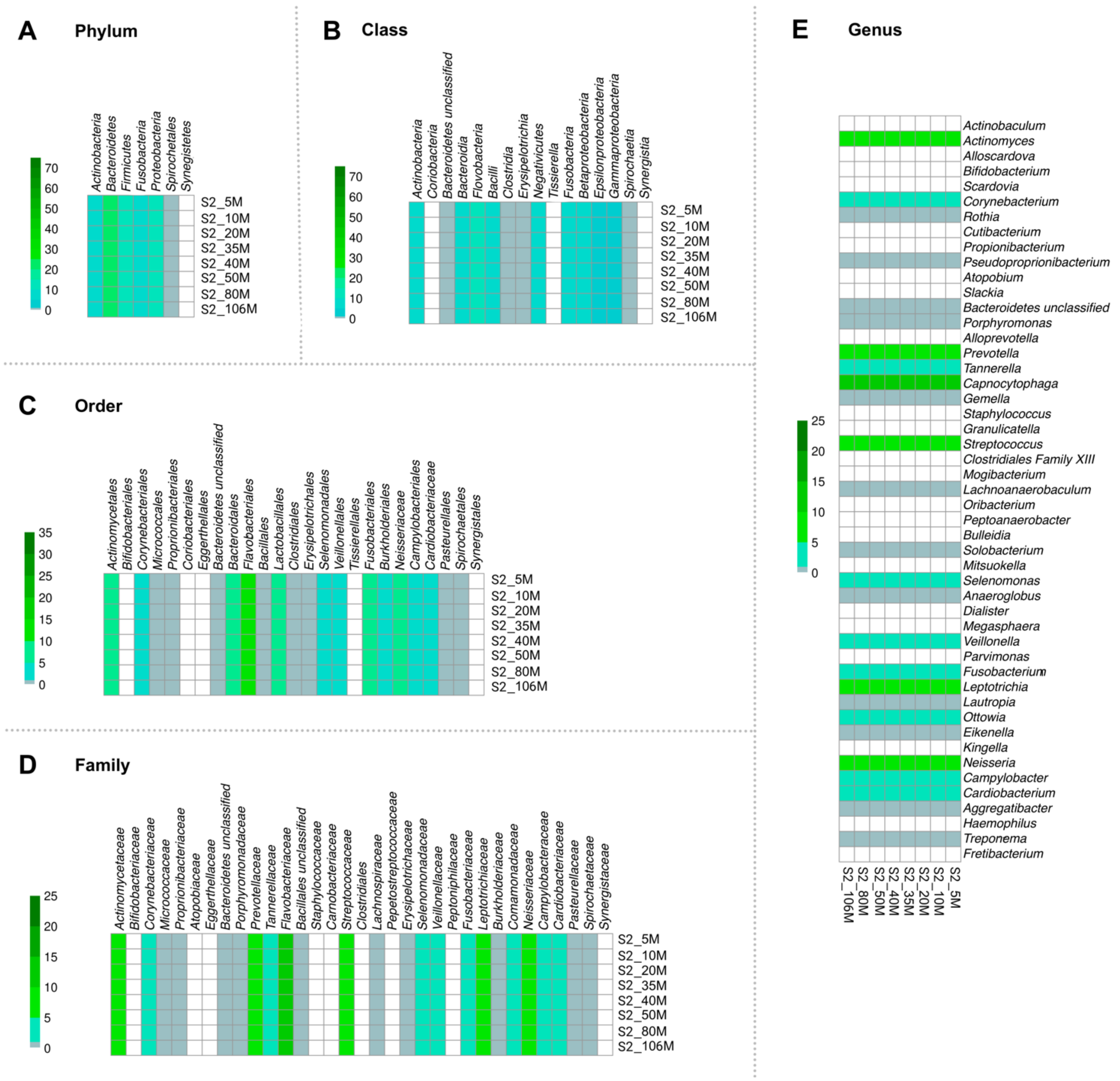

Supplementary Figure S2. Quantitative taxonomic profiling of S2 datasets at different taxonomic levels: **A** *Phylum*, **B** Class, **C** Order, **D** Family, and **E** *Genus*. The colored bar (grey to dark green) indicates percent abundance.

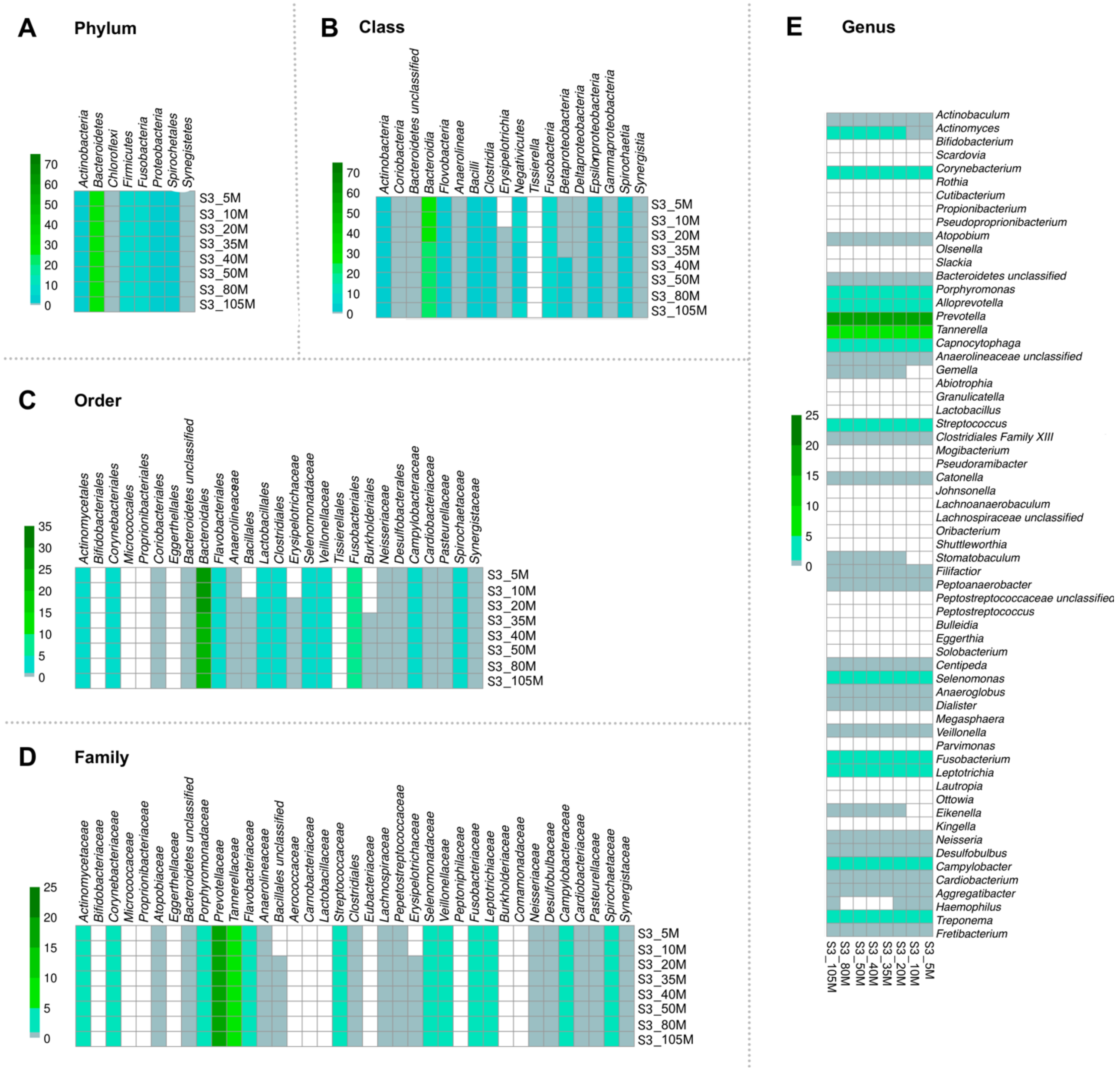

Supplementary Figure S3. Quantitative taxonomic profiling of S3 datasets at different taxonomic levels: **A** *Phylum*, **B** Class, **C** Order, **D** Family, and **E** *Genus*. The colored bar (grey to dark green) indicates percent abundance.

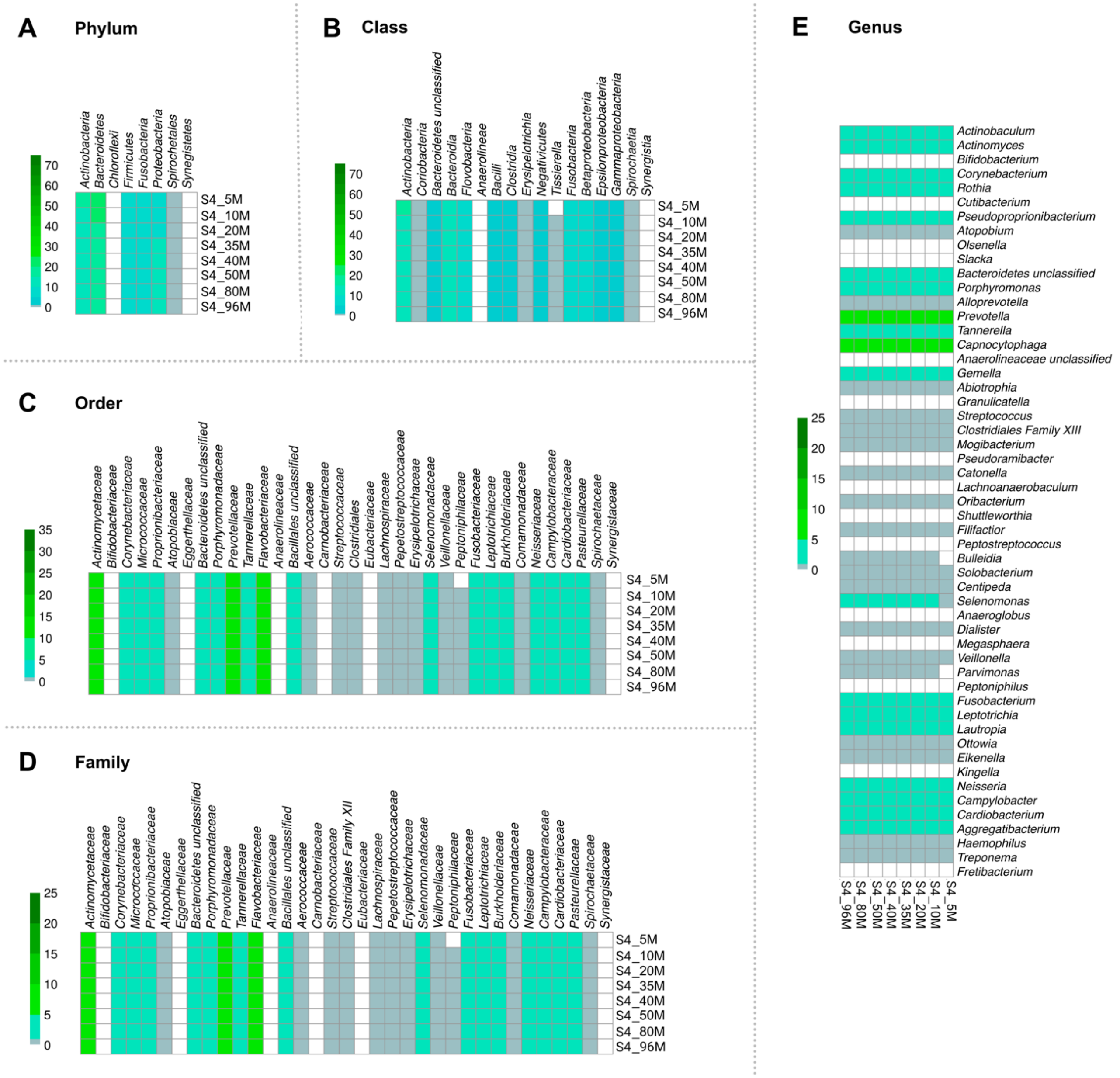

Supplementary Figure S4. Quantitative taxonomic profiling of S4 datasets at different taxonomic levels: **A** *Phylum*, **B** Class, **C** Order, **D** Family, and **E** *Genus*. The colored bar (grey to dark green) indicates percent abundance.

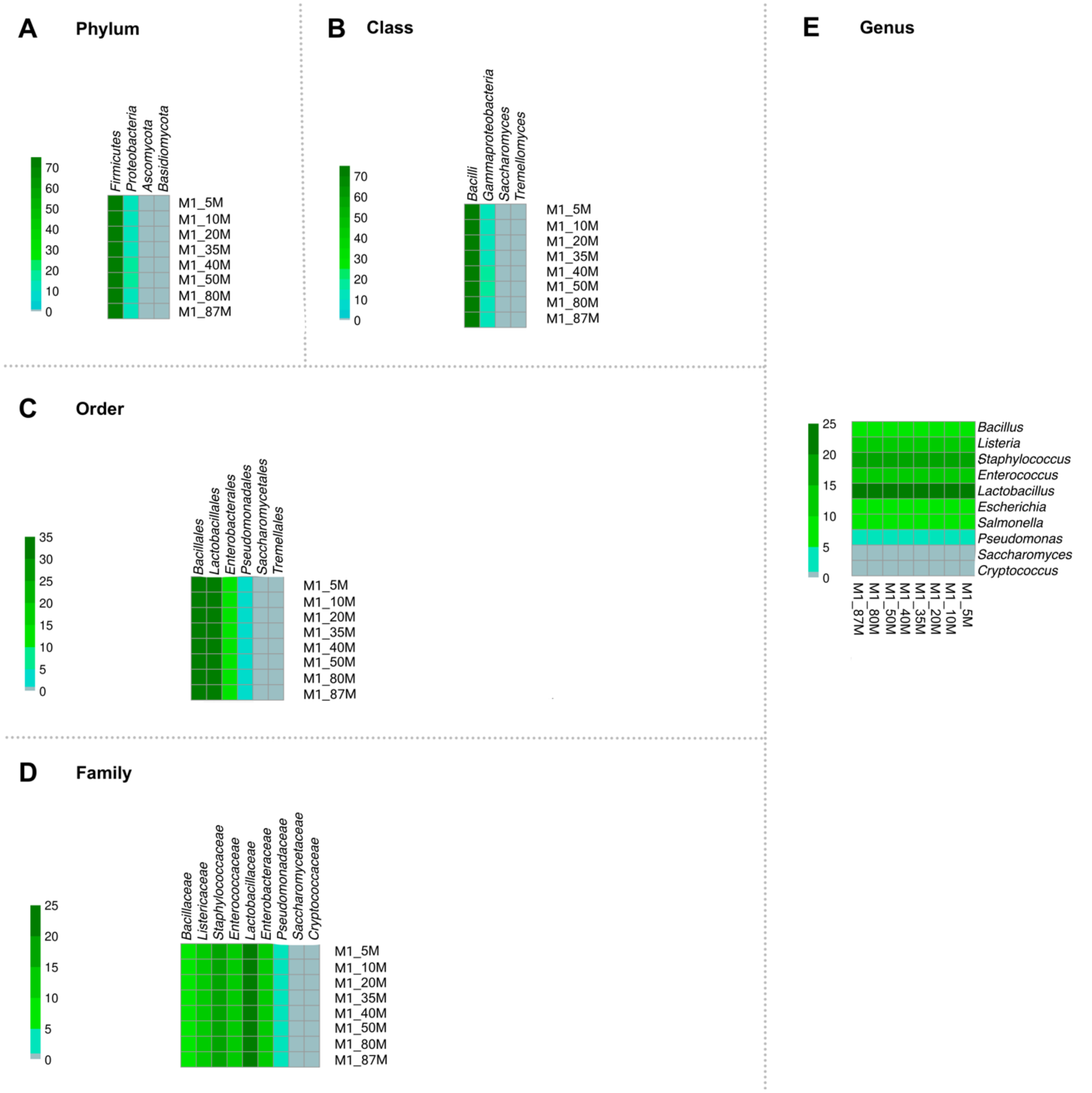

Supplementary Figure S5. Quantitative taxonomic profiling of M1 datasets at different taxonomic levels: **A** *Phylum*, **B** Class, **C** Order, **D** Family, and **E** *Genus*. The colored bar (grey to dark green) indicates percent abundance.

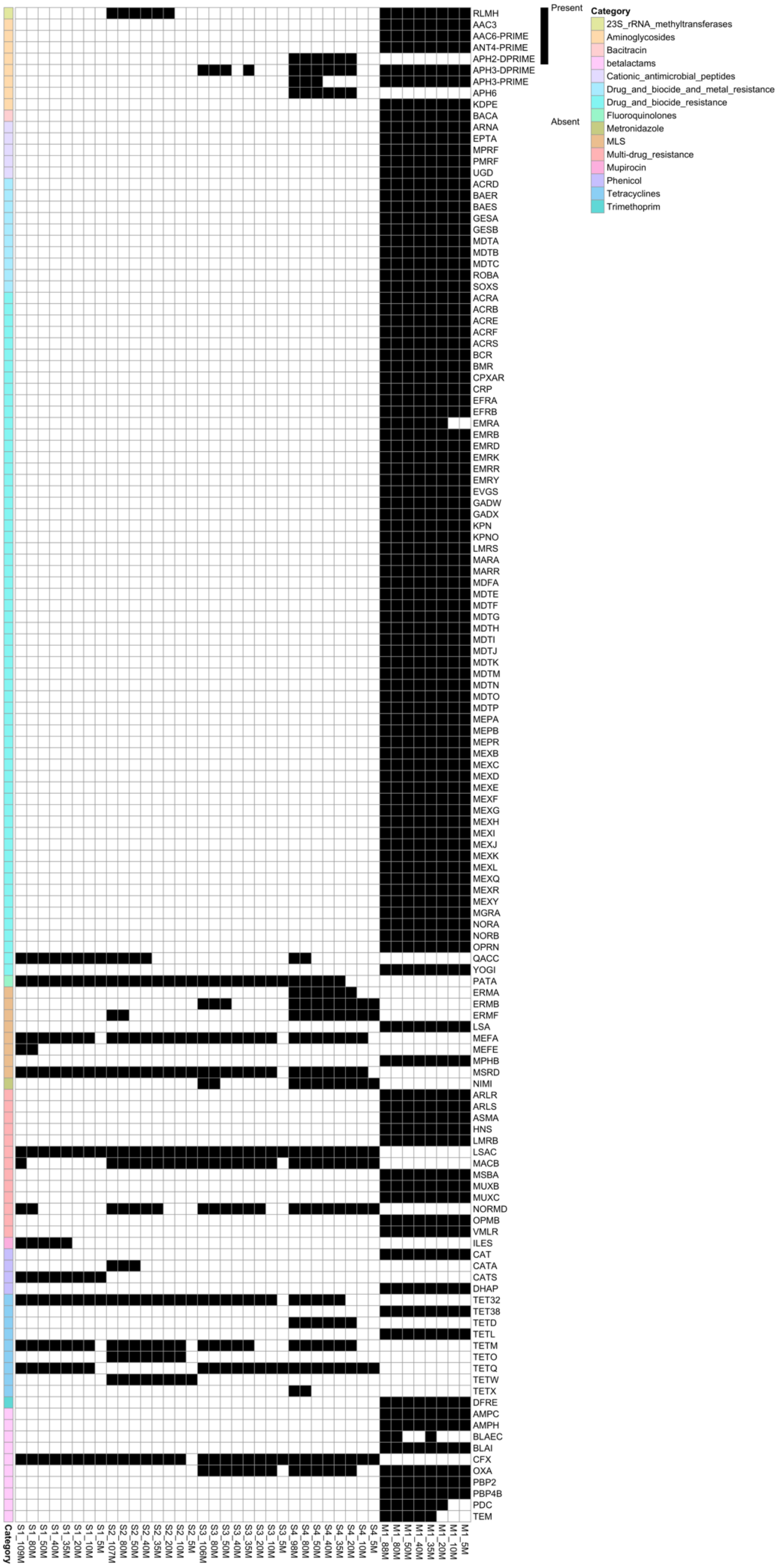
RLMH
AAC3
AAC6-PRIME
ANT4-PRIME
APH2-DPRIME
APH3-DPRIME
APH3-PRIME
APH6
KDPE
BACA
ARNA
EPTA
MPRF
PMRF
UGD
ACRD
BAER
BAES
GESA
GESB
MDTA
MDTB
MDTC
ROBA
SOXS
ACRA
ACRB
ACRE
ACRF
ACRS
BCR
BMR
CPXAR
CRP
EFRA
EFRB
EMRA
EMRB
EMRD
EMRK
EMRR
EMRY
EVGS
GADW
GADX
KPN
KPNO
LMRS
MARA
MARR
MDFA
MDTE
MDTF
MDTG
MDTH
MDTI
MDTJ
MDTK
MDTM
MDTN
MDTO
MDTP
MEPA
MEPB
MEPR
MEXB
MEXC
MEXD
MEXE
MEXF
MEXG
MEXH
MEXI
MEXJ
MEXK
MEXL
MEXQ
MEXR
MEXY
MGRA
NORA
NORB
OPRN
QACC
YOGI
PATA
ERMA
ERMB
ERMF
LSA
MEFA
MEFE
MPHB
MSRD
NIMI
ARLR
ARLS
ASMA
HNS
LMRB
LSAC
MACB
MSBA
MUXB
MUXC
NORMD
OPMB
VMLR
ILES
CAT
CATA
CATS
DHAP
TET32
TET38
TETD
TETL
TETM
TETO
TETQ
TETW
TETX
DFRE
AMPC
AMPH
BLAEC
BLAI
CFX
OXA
PBP2
PBP4B
PDC
TEM
Category
S1_109M
S1_80M
S1_50M
S1_40M
S1_35M
S1_20M
S1_10M
S1_5M
S2_107M
S2_80M
S2_50M
S2_40M
S2_35M
S2_20M
S2_10M
S2_5M
S3_106M
S3_80M
S3_50M
S3_40M
S3_35M
S3_20M
S3_10M
S3_5M
S4_98M
S4_80M
S4_50M
S4_40M
S4_35M
S4_20M
S4_10M
S4_5M
M1_88M
M1_80M
M1_50M
M1_40M
M1_35M
M1_20M
M1_10M
M1_5M
Present
Absent
Category
23S_rRNA_methyltransferases
Aminoglycosides
Bacitracin
betalactams
Cationic_antimicrobial_peptides
Drug_and_biocide_and_metal_resistance
Drug_and_biocide_resistance
Fluoroquinolones
Metronidazole
MLS
Multi-drug_resistance
Mupirocin
Phenicol
Tetracyclines
Trimethoprim

Supplementary Figure S6. Antimicrobial resistance genes (ARGs) presence/absence heatmap. Only ARGs with >50% of nucleotides covered by at least one read were defined as present in the sample. MLS= Macrolides, Lincosamides, Streptogramines.
